# A destabilizing Y891D mutation in activated EGFR impairs sensitivity to kinase inhibition

**DOI:** 10.1038/s41698-023-00490-w

**Published:** 2024-01-05

**Authors:** Daniel S. Lenchner, Zaritza O. Petrova, Lisa Hunihan, Kumar D. Ashtekar, Zenta Walther, Frederick H. Wilson

**Affiliations:** 1 Department of Internal Medicine, Section of Medical Oncology, Yale School of Medicine, New Haven, CT USA; 2 Department of Genetics, Yale School of Medicine, New Haven, CT USA; 3 Center of Molecular and Cellular Oncology, Yale Cancer Center, Yale School of Medicine, New Haven, CT USA; 4 Department of Pharmacology, Yale School of Medicine, New Haven, CT USA; 5 Yale Cancer Biology Institute, Yale University West Campus, West Haven, CT USA; 6 Department of Pathology, Yale School of Medicine, New Haven, CT USA

**Keywords:** Translational research, Non-small-cell lung cancer

## Abstract

EGFR tyrosine kinase inhibitors (TKIs) have transformed the treatment of EGFR-mutated non-small cell lung carcinoma (NSCLC); however, therapeutic resistance remains a clinical challenge. Acquired secondary EGFR mutations that increase ATP affinity and/or impair inhibitor binding are well-described mediators of resistance. Here we identify a de novo EGFR Y891D secondary alteration in a NSCLC with EGFR L858R. Acquired EGFR Y891D alterations were previously reported in association with resistance to first generation EGFR TKIs. Functional studies in Ba/F3 cells demonstrate reduced TKI sensitivity of EGFR L858R + Y891D, with the greatest reduction observed for first and second generation TKIs. Unlike other EGFR mutations associated with TKI resistance, Y891D does not significantly alter ATP affinity or promote steric hindrance to inhibitor binding. Our data suggest that the Y891D mutation destabilizes EGFR L858R, potentially generating a population of misfolded receptor with preserved signaling capacity but reduced sensitivity to EGFR inhibitors. These findings raise the possibility of protein misfolding as a mechanism of resistance to EGFR inhibition in EGFR-mutated NSCLC.

## Introduction

Lung cancer remains the leading cause of cancer-related deaths worldwide^1^. Activating mutations in the epidermal growth factor receptor (EGFR) occur in 15-20% of lung adenocarcinomas in Western populations^2^. The most common activating EGFR alterations in non-small cell lung carcinoma (NSCLC) are somatic in-frame deletions within exon 19 and the L858R missense mutation in exon 21, together comprising 80–90% of activating EGFR mutations^2,3^. For nearly 20 years, EGFR-directed tyrosine kinase inhibitors (TKIs) have been the mainstay of therapies for advanced EGFR-mutated NSCLC.

Although most patients benefit from EGFR TKIs, drug resistance remains a major clinical challenge as most patients eventually experience NSCLC progression that limits the durability of these therapies. Reported clinical mechanisms of resistance to EGFR inhibitors are diverse and include on-target secondary EGFR mutations, activation of bypass pathways, and histologic transformation^4–6^. The secondary EGFR mutation T790M is identified in approximately half of tumors from patients with acquired resistance to first generation ATP-competitive EGFR inhibitors and promotes resistance both by increasing EGFR affinity for ATP and impairing inhibitor binding^7–10^. The third generation EGFR inhibitor osimertinib was developed to overcome the effects of the EGFR T790M mutation and demonstrates superior activity and improved clinical outcomes compared to earlier generation inhibitors^5,11–13^. As a result, osimertinib is currently the standard first-line treatment for patients with advanced EGFR-mutated NSCLC in the United States.

Activating EGFR mutations are associated with varying degrees of sensitivity to EGFR inhibitors. For example, patients with NSCLC harboring EGFR L858R or atypical activating mutations have shorter progression-free survival with osimertinib compared to patients whose tumors have the classic EGFR ∆E746-A750 exon 19 deletion^12,14^. More recent data identified a rare exon 19 deletion with introduction of a proline at position 747 (∆L747-A750InsP) with increased ATP affinity that shows reduced sensitivity to first and third generation EGFR inhibitors^15–17^. In addition, de novo EGFR mutations that co-occur with classic activating EGFR alterations and impact sensitivity to EGFR inhibition have been described in some cases. For instance, the EGFR resistance mutation T790M can occur as a de novo alteration in conjunction with activating EGFR mutations and is associated with primary resistance to first generation EGFR inhibitors^18,19^. EGFR G796D is also described in a case report as a de novo alteration associated with resistance to osimertinib^20^.

Here we report a de novo somatic EGFR Y891D alteration in a patient with advanced NSCLC harboring EGFR L858R with a best response of stable disease when treated with osimertinib. EGFR Y891D (corresponding to Y867 in mature EGFR numbering without the 24 residues of the signal peptide) has been reported previously in EGFR-mutated NSCLC as an acquired alteration associated with clinical resistance to first generation EGFR inhibitors^21,22^. However, functional characterization of this mutation has not been described. We note that Y891 is located more distally than other EGFR resistance alterations, in exon 22 between helices αEF and αF in the C-lobe of the tyrosine kinase domain (TKD) and far (>18 Å) from the substrate binding site. Given the unusual location of this alteration and its potential association with clinical resistance, we performed cellular and biochemical studies to characterize the EGFR Y891D mutation.

## Results

### Co-occurrence of EGFR L858R and Y891D in a patient with advanced NSCLC

**Fig. 1:**
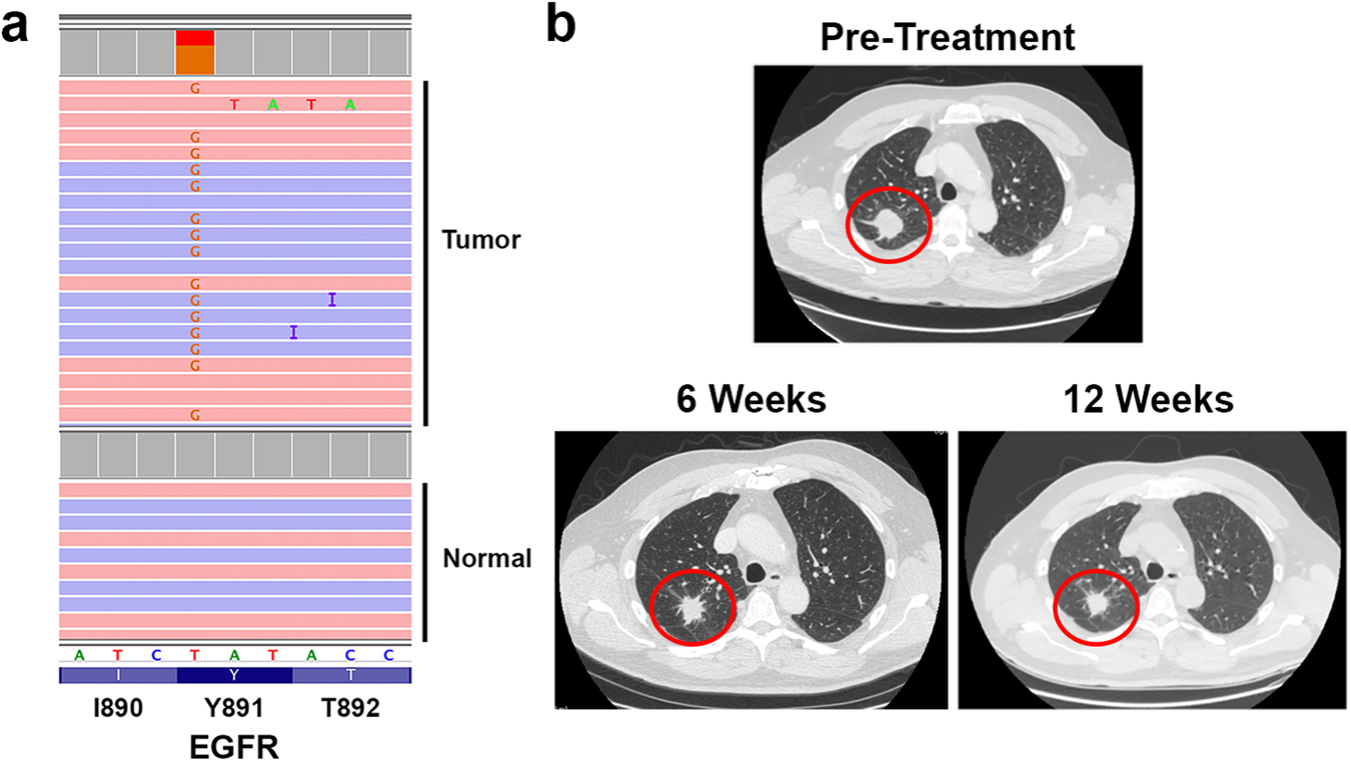
Identification of EGFR Y891D in a patient with advanced NSCLC with EGFR L858R. **a** Tumor molecular profiling identified EGFR Y891D from a resected brain metastasis prior to initiation of systemic therapy. Pink and blue bars represent forward and reverse sequencing reads from tumor and normal germline control. Data are displayed using the Integrative Genomics Viewer. **b** Computed tomography (CT) imaging of the chest demonstrating the primary lung mass prior to treatment and after 6 weeks and 12 weeks of osimertinib therapy. After 6 weeks of osimertinib, the tumor decreased in size from 3.2 to 2.6 cm (−18.75%), consistent with stable disease by RECIST criteria. There was no further change in size after an additional 6 weeks of treatment.

A 62 year-old Caucasian male with no prior smoking history presented with ongoing headache for 4 weeks. Head CT and subsequent brain MRI demonstrated a 3.5 cm cerebellar lesion causing mass effect. CT imaging of the body revealed a 3.2 cm right upper lobe lung mass and a 1.1 cm right hilar lymph node. The patient underwent left suboccipital craniectomy with resection of the cerebellar lesion. Pathologic evaluation demonstrated metastatic lung adenocarcinoma (LUAD). Tumor PD-L1 staining was positive in 60% of tumor cells. Tumor molecular profiling demonstrated an activating EGFR L858R mutation and an EGFR Y891D mutation at similar allele fractions (Fig. 1a, Table 1). Both L858R and Y891D were somatic alterations absent from the germline. Other identified variants are shown in Table 1. No molecular alterations were detected from circulating tumor DNA (ctDNA) analysis of a peripheral blood sample, likely due to limited systemic tumor burden. PET-CT demonstrated hypermetabolism of the lung mass with no evidence of mediastinal or distant metastases. Mediastinal lymph node sampling via cervical mediastinoscopy showed no evidence of tumor involvement.

**Table 1:** Genetic alterations in pre- and post-treatment samples.

Pre-treatment (40% Tumor)		Post-treatment (60% Tumor)	
Alteration	Allele frequency (%)	Alteration	Allele frequency (%)
EGFR L858R	47	EGFR L858R	65
EGFR Y891D	46	EGFR Y891D	64
TP53 D259V	59	TP53 D259V	60
BAP1 R592I	18	NOTCH2 E2266K	12
RAD50 E431K	15	PMS2 L266V	3
DDR2 F764V	14		
SETD2 Q1215E	14		

Pre- and post-treatment tumor samples obtained from brain and adrenal, respectively.

Systemic therapy with osimertinib was initiated. Repeat chest CT after 6 weeks of therapy showed a modest reduction in size of the lung tumor from 3.2 to 2.6 cm, consistent with stable disease by Response Evaluation Criteria in Solid Tumors (RECIST); subsequent imaging an additional 6 weeks later showed no further change in tumor size (Fig. 1b). Local therapy with radiation or surgery has been shown to improve progression-free and overall survival for patients with oligometastatic NSCLC that did not progress after 3 months of first-line systemic therapy^23^. In the setting of oligometastatic NSCLC with no radiographic evidence of other involved sites after 12 weeks of osimertinib therapy, resection of the primary tumor with right upper lobectomy was performed. Pathologic examination revealed a 2.5 cm moderately differentiated adenocarcinoma with tumor necrosis. There was no evidence of malignancy in 5 examined right hilar and mediastinal lymph node stations. Osimertinib was continued after surgery. After a total of 30 months of osimertinib therapy, the patient developed a right adrenal mass. Biopsy confirmed recurrent LUAD with redemonstration of the original EGFR L858R and Y891D mutations at similar allele fractions (Table 1). Additional variants identified in the post-treatment sample are shown in Table 1. No putative genetic mechanisms of resistance to osimertinib were identified, and there was no evidence of histologic transformation. The patient subsequently developed multiple brain and leptomeningeal metastases and rapidly succumbed to his malignancy.

### Additional clinical reports of EGFR Y891D

Two prior case reports identified an acquired EGFR Y891D alteration in the setting of resistance to first generation EGFR TKIs, although no functional studies were described^21,22^. The first report identified Y891D in an advanced LUAD with EGFR L858R from a patient who had progressed on the first generation EGFR inhibitor gefitinib^21^. The patient was subsequently treated with osimertinib with stable disease of unspecified duration. Based on structural modeling studies, the authors of this study suggested that Y891D increases ATP affinity of EGFR harboring L858R to promote the apparent resistance to the first generation TKI. The second case report describes a patient with advanced squamous cell lung carcinoma with EGFR L858R and A859S who developed resistance to the first generation EGFR inhibitor icotinib^22^. Analysis of ctDNA at resistance demonstrated the presence of both the T790M and Y891D mutations. The patient was subsequently treated with osimertinib with clinical benefit for 8 months before developing NSCLC progression. At the time of resistance to osimertinib, repeat ctDNA analysis showed an increased allele fraction of Y891D and decreased allele fraction of L858R and A859S, while T790M was no longer detectable. The patient was subsequently treated with afatinib with clinical deterioration within 2 months.

We queried the AACR Project Genie database (Cohort v14.0-public dataset) in search of other examples of EGFR Y891D^24^. From 160,965 patients, we identified 25,235 patients with a diagnosis of non-small cell lung carcinoma. Among these, we found 4771 patients whose tumor had an EGFR mutation, including 1474 with EGFR L858R. A de novo EGFR Y891D alteration was identified in one patient (GENIE-MSK-P-0016613) with early-stage LUAD with EGFR L858R reported in a next-generation sequencing study of treatment-naïve LUAD patients^25^. EGFR Y891D was not identified in any of the other tumors in the database. We conclude that EGFR Y891D is a rare alteration that to date has only been reported in conjunction with an activating EGFR L858R alteration.

### EGFR Y891D impairs sensitivity to EGFR inhibitors in cell-based studies

**Fig. 2:**
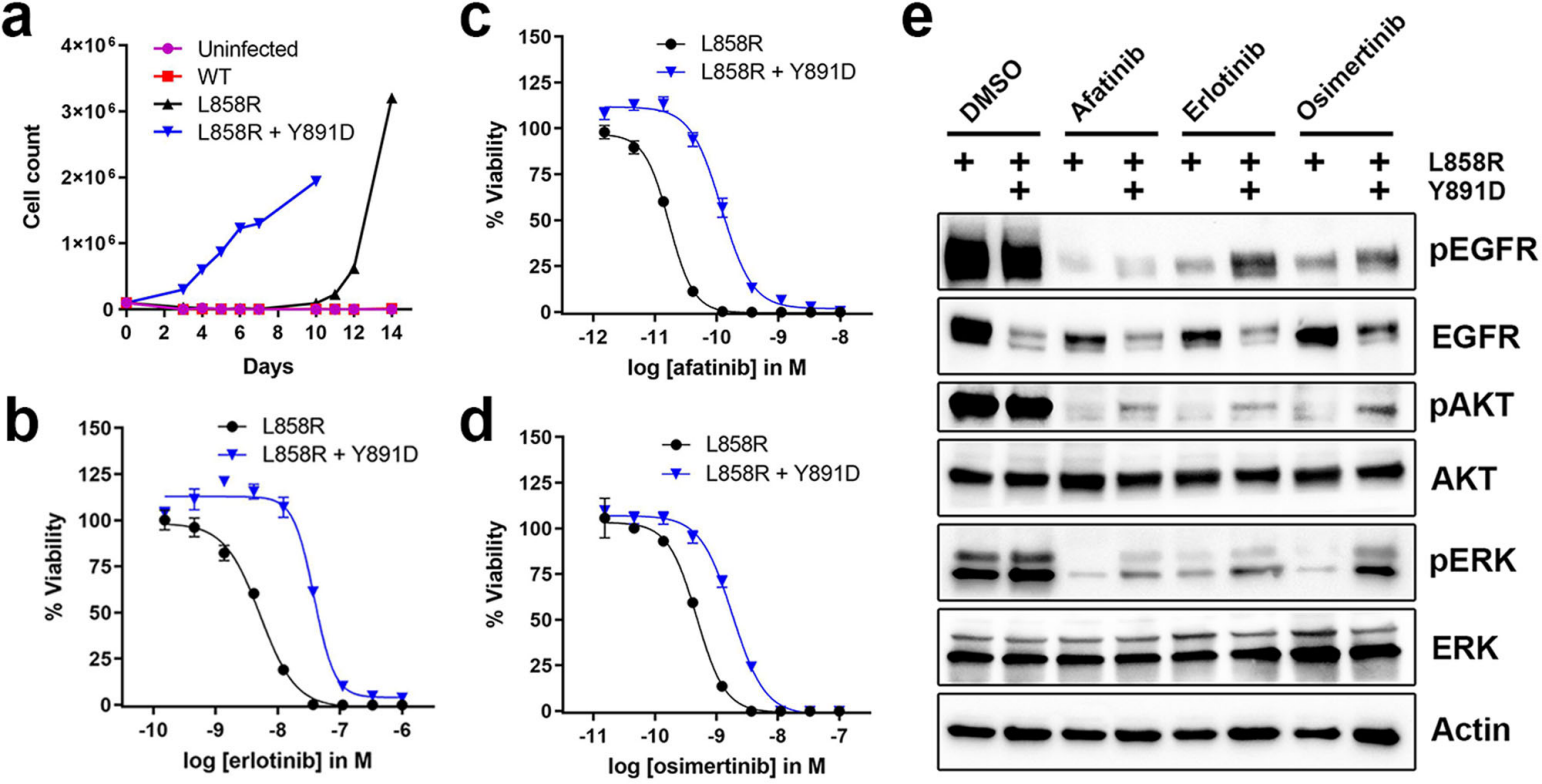
Sensitivity to EGFR inhibitors of Ba/F3 cells expressing EGFR L858R or L858R + Y891D. **a** Proliferation of uninfected Ba/F3 cells or Ba/F3 cells expressing wild-type (WT) EGFR, EGFR L858R, or EGFR L858R + Y891D after withdrawal of IL-3 (Day 0). **b** Ba/F3 cells expressing EGFR L858R or EGFR L858R + Y891D were exposed to erlotinib at the indicated concentrations. After 3 days, cell viability was determined using Cell Titer-Glo. Mean and SEM is shown, and the experiment was performed 4 times. **c** As in **b**, except with afatinib. **d** As in **b**, except with osimertinib. **e** Western immunoblotting of lysates from Ba/F3 cells expressing EGFR L858R or EGFR L858R + Y891D treated with DMSO, afatinib (10 nM), erlotinib (100 nM), or osimertinib (50 nM) for 4 h.

Given the potential impact of EGFR Y891D on sensitivity of activated EGFR to inhibition and its unusual location distal to the substrate binding site and other resistance mutations in the EGFR TKD, we undertook functional and biochemical characterization of this secondary alteration. We introduced EGFR with L858R alone or L858R + Y891D into the interleukin-3 (IL-3)–dependent murine Ba/F3 hematopoietic cell line. IL-3 was withdrawn from the culture medium to generate Ba/F3 cells that were dependent on mutated EGFR. Like those expressing EGFR L858R (but not those with wild-type EGFR), Ba/F3 cells expressing EGFR L858R + Y891D proliferated in the absence of IL-3 (Fig. 2a). EGFR with Y891D alone was not evaluated as Y891D is not an activating mutation (see below). We next compared the sensitivity of Ba/F3 cells expressing EGFR L858R or L858R + Y891D to EGFR TKIs in cell viability assays. Introducing the Y891D mutation alongside L858R caused an approximately 7-fold increase in half-maximal inhibitory concentration (IC_50_) for the first generation EGFR inhibitor erlotinib (Fig. 2b) and the second generation inhibitor afatinib (Fig. 2c). An increase in IC_50_ was also observed with osimertinib, but the difference was more modest at approximately 3.5-fold (Fig. 2d). Immunoblotting studies demonstrated that Ba/F3 cells expressing EGFR L858R + Y891D showed persistent activation of EGFR and downstream RAF-MEK-ERK and PI3K pathways in the presence of EGFR inhibitors compared to EGFR L858R, despite substantially lower total EGFR levels than seen for EGFR L858R (Fig. 2e). These cell-based results therefore indicate that introducing the Y891D mutation into L858R-mutated EGFR substantially reduces sensitivity to erlotinib and afatinib, with a smaller effect on osimertinib sensitivity.

### Biochemical comparison of L858R-mutated and L858R + Y891D-mutated EGFR in vitro

We next tested the hypothesis that Qin et al. proposed based on their computational analysis that the Y891D mutation increases ATP-binding affinity of EGFR to reduce sensitivity to ATP-competitive inhibitors (as seen for other EGFR resistance mutations)^8,16,21^. We generated baculovirus expression constructs for relevant variants of the isolated EGFR TKD and purified the TKD for kinase assays as described^16^. We observed that expression of EGFR-TKD harboring the L858R + Y891D compound mutation (EGFR-TKD^L858R + Y891D^) was substantially reduced compared with EGFR-TKD^Y891D^ (Supplementary Fig. 1a) or with wild-type or L858R-mutated TKD, consistent with the reduced expression of the full-length L858R + Y891D EGFR variant observed in Ba/F3 cells (Fig. 2e). We were nonetheless able to generate sufficient high quality purified EGFR-TKD^L858R + Y891D^ for kinase activity measurements (Supplementary Fig. 1b–d) but found that the protein rapidly lost activity (within approximately 1 h).

**Fig. 3:**
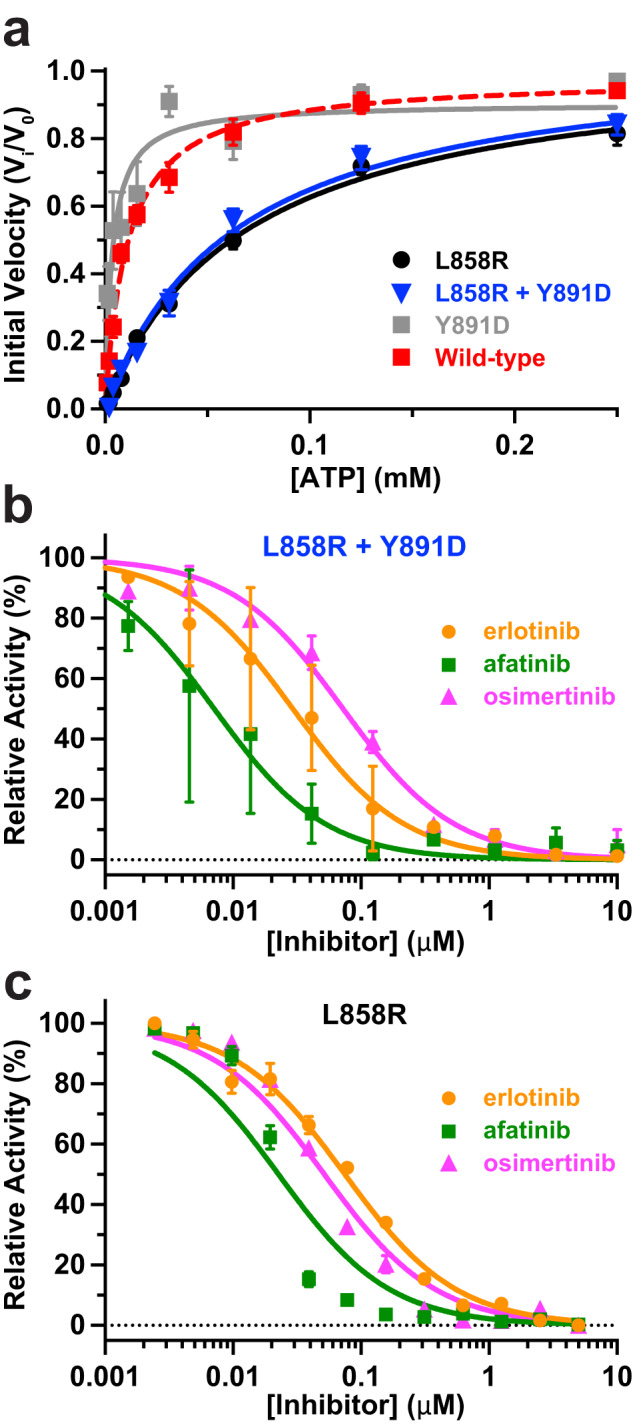
Comparison of *K*_M, ATP_ and inhibitor sensitivity for EGFR-TKD^L858R + Y891D^ and EGFR-TKD^L858R^. **a** Activity (initial velocity) as a function of ATP concentration for EGFR-TKD^L858R + Y891D^, EGFR-TKD^L858R^, EGFR-TKD^Y891D^ and EGFR-TKD^WT^, demonstrating that *K*_M, ATP_ is increased by the L858R mutation but not the Y891D mutation. See Table 2 for best fit *K*_M, ATP_ values. Each data point represents three biological repeats, each performed in triplicate, with error bars representing SEM. Inhibition curves used to estimate IC_50_ values for erlotinib, afatinib, and osimertinib with EGFR-TKD^L858R + Y891D^ (**b**) or EGFR-TKD^L858R^ (**c**). Best fit data are presented in Table 2 and represent two biological repeats, each performed in triplicate, with errors quoted as SEM.

Using only freshly purified protein, we measured TKD activity using a continuous fluorescence-based assay (see Methods) that follows phosphorylation of a reporter peptide containing a sulfonamido-oxine (Sox) fluorophore^26^. We found that EGFR-TKD^Y891D^ has very similar activity (within 3-4 fold) to the wild-type TKD (Table 2), whereas EGFR-TKD^L858R + Y891D^ is >10-fold more active, reflecting the known activating effect of the L858R mutation^27^. These data indicate that EGFR Y891D itself is not an activating mutation. We next measured kinase activity as a function of ATP concentration (Fig. 3a) to estimate Michaelis constants for ATP (*K*_M, ATP_), which are reduced in inhibitor-resistant T790M and in a subset of exon 19 mutations because of increased ATP-binding affinity^8,16^. The known ~6-fold increase in *K*_M, ATP_ reported for the L858R mutation^8^ is retained in EGFR-TKD^L858R + Y891D^ (Fig. 3a, Table 2). In the context of an otherwise wild-type TKD, the Y891D mutation does appear to reduce *K*_M, ATP_ slightly (by 3.3-fold, *P* = 0.002; Fig. 3a), but the *K*_M, ATP_ value for EGFR-TKD^L858R + Y891D^ appears to be driven primarily by the known effects of the L858R mutation. This result suggests that the reduced TKI sensitivity observed for EGFR with the L858R + Y891D mutations does not reflect an increased affinity for ATP (in contrast with T790M, for example).

**Table 2:** Enzymatic and inhibition parameters for EGFR kinase domain variants.

Mutation	*K*_M, ATP_ (μM)	*K*_M, pept_^b^ (μM)	*k*_cat_ (app)^b^ (s^−1^)	erlotinib IC_50_ (nM)	afatinib IC_50_ (nM)	osimertinib IC_50_ (nM)
None (WT)^a^	12 ± 1	406	0.042	n.d.	n.d.	n.d.
L858R^a^	74 ± 5	218	1.4	75 ± 5	23 ± 1	52 ± 1
L858R + Y891D	76 ± 11	447	2.8	39 ± 28	12 ± 10	72 ± 3
Y891D	3.6 ± 2	1265	0.15	n.d.	n.d.	n.d.

*K*_M, ATP_ values are reported as mean ± SEM of 3 independent biological experiments performed in triplicate. IC_50_ measurements are reported as mean ± SEM of two independent biological experiments, each performed in triplicate.

^a^Data for the WT TKD and L858R TKDs are as previously reported^16^.

^b^Titration to maximum peptide concentration was only performed once because of the large amount of peptide required; thus, *K*_M, pept_ and apparent *k*_cat_ values are estimates with no SEM quoted (*n* = 1).

We next measured IC_50_ values for in vitro inhibition of EGFR TKD by the inhibitors studied in Fig. 2. IC_50_ values were unexpectedly not significantly different between EGFR-TKD^L858R^ and EGFR-TKD^L858R + Y891D^ for erlotinib (*P* = 0.33) or afatinib (*P* = 0.39) and trended slightly lower for EGFR-TKD^L858R + Y891D^ in both cases (Table 2, Fig. 3b, c). This lack of a significant IC_50_ difference is consistent with our analysis of *K*_M, ATP_ and *k*_cat_, however. A slight (~1.4-fold) reduction in sensitivity to osimertinib (*P* = 0.02) was seen for EGFR-TKD^L858R + Y891D^ compared with EGFR-TKD^L858R^.

### Y891D destabilizes EGFR L858R

Both biochemical and cellular analyses of EGFR L858R + Y891D demonstrated that introducing a Y891D mutation into the L858R-mutated variant of EGFR has a substantial negative impact on its stability, as assessed by relative expression levels (Fig. 2e and Supplementary Fig. 1a). Importantly, the Y891D mutation alone does not have this effect when introduced into the wild-type TKD (Supplementary Fig. 1a). Moreover, our in vitro studies of EGFR-TKD^L858R + Y891D^ demonstrated that kinase activity was rapidly lost (within 1 h), presumably due to denaturation and precipitation of the purified enzyme. In assays performed within the first hour after purification, however, measurable parameters for EGFR-TKD ^L858R + Y891D^ were essentially the same as those for EGFR-TKD ^L858R^. These observations led us to investigate the possible origin of the loss in stability and whether it might underlie the reduced inhibitor sensitivity in cellular studies and in patients.

**Fig. 4:**
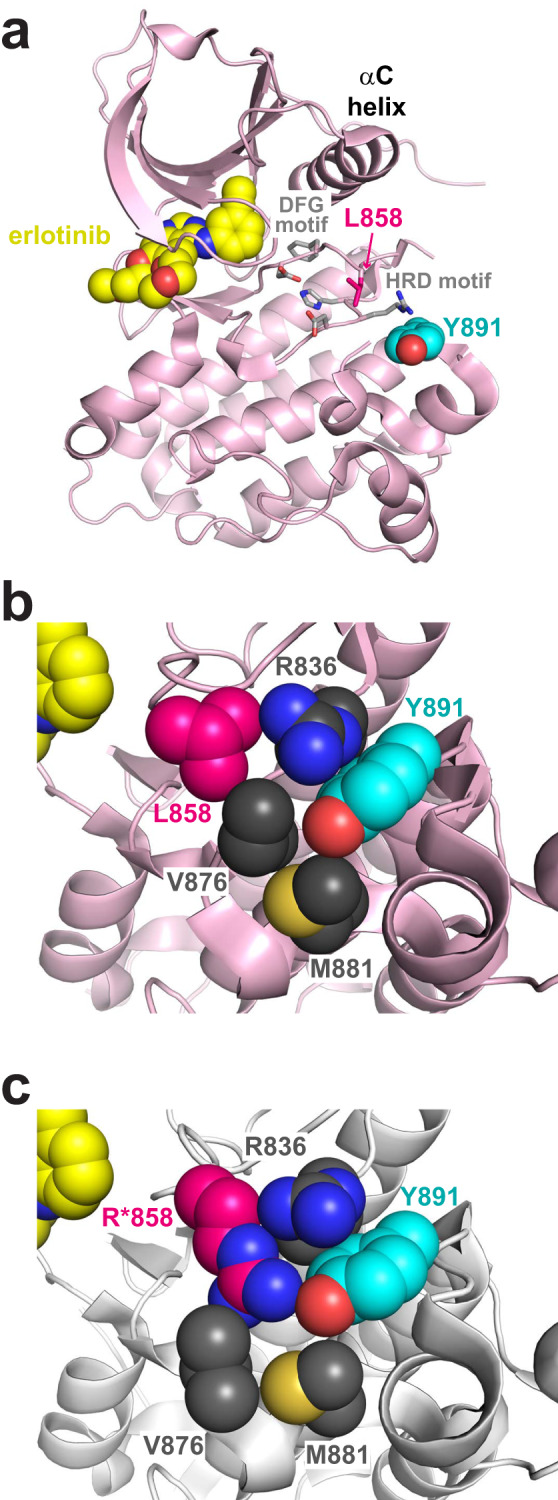
Possible role for Y891 in stabilizing EGFR with L858R. **a** Ribbon representation of the wild-type EGFR TKD bound to erlotinib (from PDB ID 1M17). EGFR-TKD^WT^ is in light pink, and erlotinib is shown in yellow spheres. The positions of Y891 (~20 Å from the ATP-binding site) and L858 are shown, with the residues colored respectively in cyan and magenta. The conserved kinase HRD and DFG motifs are also labeled in gray, with relevant side-chains shown. **b** Close-up view of how Y891 is accommodated in the wild-type kinase (from PDB ID 1M17). The Y891 side-chain nestles in a pocket formed largely by the R836, V876, and M881 side-chains, with L858 in the ‘second shell’ of surrounding side-chains. **c** When L858 is mutated to arginine, the R858 side-chain penetrates the pocket used by Y891 and interacts with it directly (from PDB ID 2EB3).

The Y891 side-chain is some distance (>18 Å) away from erlotinib in the ATP-binding site of the wild-type EGFR TKD (Fig. 4a). Consistent with this, our kinase assays showed that the Y891D mutation does not substantially affect ATP binding (Table 2). In crystal structures of the wild-type TKD in the Protein Data Bank (PDB; e.g. PDB ID 1M17)^28^, the Y891 side-chain lies in a pocket (Fig. 4b) that comprises the side-chains from R836 (from the conserved HRD motif of the kinase), V876, and M881 with the L858 side-chain packing against both R836 and V876 on the outside of this pocket. When L858 (magenta in Fig. 4b) is mutated to arginine in the most common activating NSCLC variant (L858R), the introduced arginine side-chain must penetrate this pocket, and its guanidino group comes within 2.7 Å (in PDB ID 2EB3)^29^ to 3.6 Å (PDB ID 5CZH)^30^ of the tyrosine hydroxyl of Y891 in different structures of the L858R-mutated TKD in the PDB (Fig. 4c). These observations suggest that the nature and location of the Y891 side-chain may be important for stabilizing the L858R-mutated EGFR TKD. The side-chain of an aspartate that replaces Y891 will be >5 Å away from the arginine at position 858, leaving the polar arginine in the mutated TKD in a largely hydrophobic pocket without a nearby counterion. This may explain our findings that the EGFR L858R and EGFR Y891D TKD variants are both well-expressed and stable, whereas folding of the compound EGFR L858R + Y891D variant is substantially compromised. Indeed, molecular dynamics (MD) simulations further showed that an aspartate at position 891 interacts primarily with R836, leaving the arginine in position 858 in L858R-mutated EGFR without an interacting polar group. This difference was also evident in separate MD simulations that showed increased dynamics in the activation loop of EGFR-TKD^L858R + Y891D^ compared with the wild-type or Y891D-mutated variants.

### A residual erlotinib-resistant population of EGFR L858R + Y891D

**Fig. 5:**
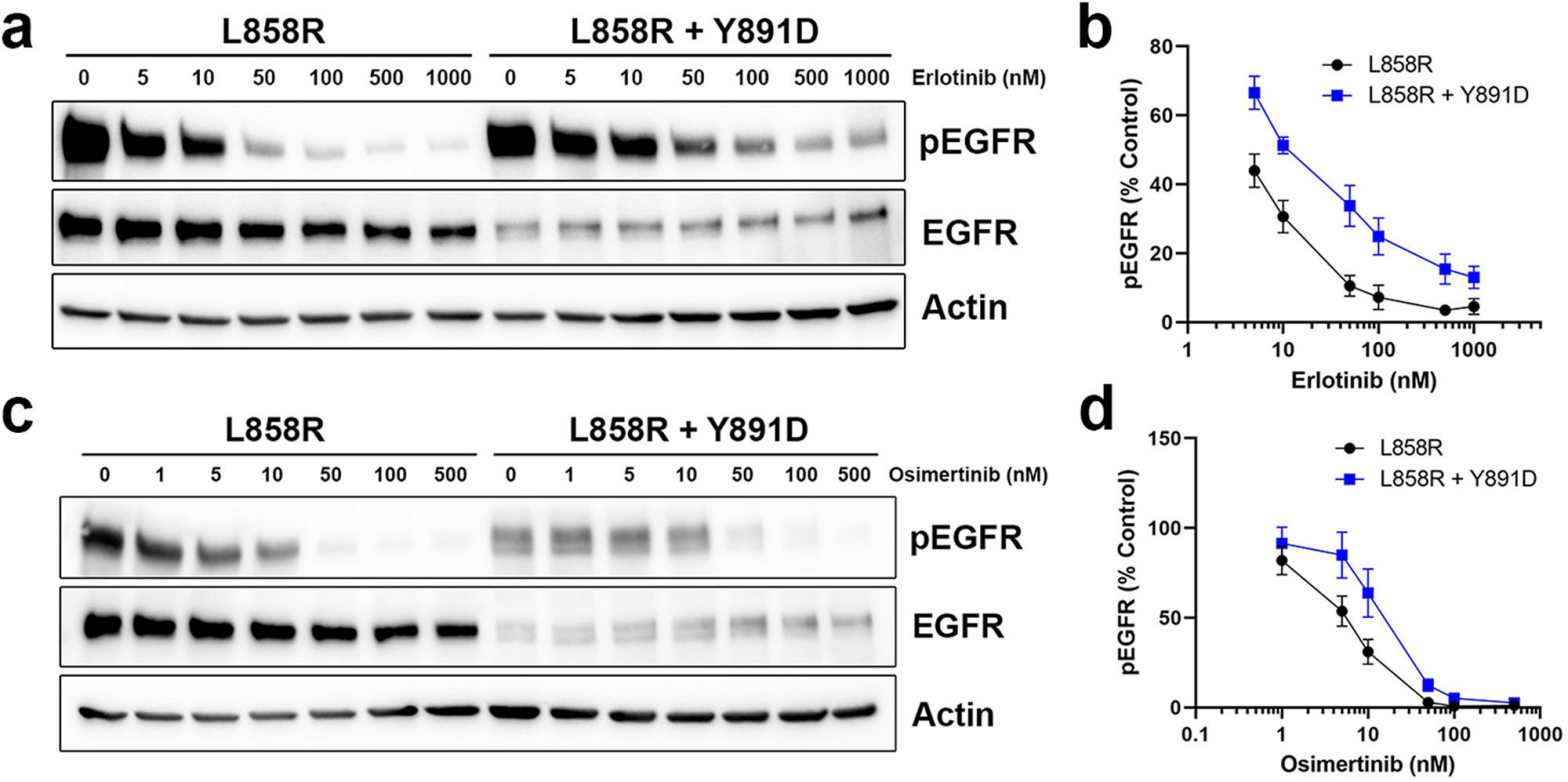
Concentration dependence of EGFR inhibition with erlotinib or osimertinib in Ba/F3 cells expressing EGFR L858R or L858R + Y891D. **a** Western immunoblotting of lysates from Ba/F3 cells expressing EGFR L858R or EGFR L858R + Y891D treated with DMSO or erlotinib at the indicated concentrations (in nM) for 4 h. **b** Phospho-EGFR band densities at the indicated erlotinib concentrations (from **a**) were quantitated with normalization to total EGFR (% Control) for EGFR L858R or L858R + Y891D. Mean and SEM of 3 independent experiments is shown. **c** As in **a**, except cells were treated with osimertinib. **d** As in **b**, except for osimertinib using data from **c**.

Although our in vitro biochemical studies showed that freshly purified EGFR-TKD^L858R + Y891D^ has similar kinetic properties and IC_50_ values as L858R-mutated TKD, the protein aggregated with time and appeared to lose both inhibitor sensitivity and activity. We therefore hypothesized that the decreased TKI sensitivity associated with the L858R + Y891D variant in our cell viability assays might reflect signaling contributions from a small amount of residual misfolded and inhibitor-resistant kinase. Indeed, it is known that even small numbers of active EGFR molecules are sufficient to drive tumor cell (and presumably Ba/F3 cell) proliferation^31,32^. When bulk EGFR phosphorylation was assessed in Ba/F3 cells expressing EGFR L858R or EGFR L858R + Y891D (Fig. 5a), the dose dependencies for inhibition by erlotinib were surprisingly similar given the viability data shown in Fig. 2b. Notably, however, a residual phospho-EGFR signal persisted for EGFR L858R + Y891D even at the highest inhibitor concentrations assayed (up to 1000 nM; Fig. 5a). Furthermore, the dose-response curves for inhibition of EGFR phosphorylation by erlotinib (Fig. 5b) show the same trend for the two variants, but with the curve for EGFR L858R + Y891D translated upwards on the vertical axis at higher inhibitor concentrations as would be expected if an inhibitor-insensitive misfolded population existed and was capable of autophosphorylation. It is clear that at least a subset of L858R + Y891D-mutated EGFR is functional receptor that is processed and trafficked properly to the plasma membrane, as evidenced by the responsiveness of some of the protein to exogenous EGF (Supplementary Fig. 2) and the fact that we can purify a small amount of the active TKD for biochemical studies. Taken together, our findings raise the possibility that a misfolded or partially folded population of EGFR L858R + Y891D exists that can signal but is insensitive to early generation EGFR TKIs, potentially providing a mechanism of resistance.

Interestingly, EGFR L858R + Y891D autophosphorylation appeared to be more effectively suppressed by osimertinib than erlotinib at higher concentrations (Fig. 5c, d), consistent with the more limited reduction in sensitivity to osimertinib observed with L858R + Y891D in our cell viability assays in Ba/F3 cells (Fig. 2d). This observation suggests that a putative misfolded EGFR L858R + Y891D population is more effectively inhibited by osimertinib, which reacts covalently with the kinase domain in a manner that does not require a fully intact ATP-binding site^33^. Indeed, the dose-response curve for EGFR L858R + Y891D treated with osimertinib (Fig. 5d) does not show the upward displacement at higher inhibitor concentrations seen with erlotinib (Fig. 5b).

## Discussion

While secondary EGFR mutations are a well-characterized mechanism of acquired resistance to EGFR inhibition in EGFR-mutated NSCLC, de novo co-occurring EGFR alterations that modulate sensitivity to TKIs have only rarely been described^18–20^. Here we report co-occurrence of EGFR L858R and Y891D in a patient with advanced NSCLC. Y891D was previously reported in two NSCLC patients with EGFR L858R and acquired resistance to first generation EGFR inhibitors^21,22^. From a search of the AACR Project Genie database, we identified a second case of de novo EGFR Y891D together with L858R among a previously reported cohort of treatment-naïve NSCLCs^25^. EGFR Y891D is a rare non-activating variant, identified in only one tumor in the AACR Project Genie database.

While we cannot confirm that EGFR L858R and Y891D occur in *cis* in our patient’s tumor (since sequencing reads do not span both alterations), our findings from cell viability assays that L858R + Y891D in *cis* promotes resistance to early generation EGFR inhibitors is consistent with prior reports of clinical resistance to first generation inhibitors caused by Y891D^21,22^. Our observation that EGFR L858R + Y891D demonstrates a more modest decrease in sensitivity to the third generation EGFR inhibitor osimertinib in cell viability assays is also consistent with the limited benefit of osimertinib noted for our patient (who had a best response of stable disease by RECIST, whereas ~70% of patients with EGFR L858R have a clinical response to first-line osimertinib)^12,34^. This observation that our patient had some initial benefit from osimertinib argues that Y891D does not cause clinical resistance to osimertinib. This is consistent with the reported clinical benefit from osimertinib in one of the previously-described patients with EGFR L858R and an acquired Y891D alteration in the setting of resistance to a first generation inhibitor who subsequently had stable disease with osimertinib^21^. The other previously-described patient also had clinical benefit from osimertinib lasting 8 months after resistance to a first generation inhibitor, but an explanation for that benefit is complicated by the finding of an acquired EGFR T790M mutation in addition to Y891D from ctDNA at the time of resistance to the first generation inhibitor^22^. Our patient did subsequently develop resistance to osimertinib with tumor recurrence in the brain and a biopsy-confirmed right adrenal metastasis. While an established resistance mechanism was not identified, it is unlikely that Y891D is the driver of resistance as the patient initially experienced NSCLC control with osimertinib despite the de novo Y891D alteration.

Impaired EGFR inhibitor sensitivity associated with the T790M mutation^8^, rare exon 19 deletions such as ∆L747-A750InsP^16^, or exon 20 insertions^35^ can all readily be recapitulated in vitro with purified TKDs (which show increased ATP-binding affinity). In contrast, our in vitro biochemical data argue that this is not the case for the Y891D mutation. Thus, our findings indicate that the apparent reduced TKI sensitivity associated with Y891D differs in origin from that observed with these other EGFR alterations. It has been reported that L858R, exon 19 deletions, and exon 20 insertions all destabilize EGFR to some extent^16,36,37^, but this effect appears much more pronounced for the L858R + Y891D compound mutation, where receptor (or TKD) levels are reduced by at least 10-fold. A portion of the L858R + Y891D-mutated EGFR reaches the cell surface in Ba/F3 cells as evidenced by its activation in response to EGF (Supplementary Fig. 2). We suggest that this EGF-regulated protein corresponds to the well-folded “bulk” population that we can purify for analysis of the recombinant TKD. This well-folded population presumably remains sensitive to EGFR TKIs when studied with in vitro kinase assays. It is also likely to underlie the dose-dependent reduction in autophosphorylation of the majority of the L858R + Y891D-mutated EGFR in Ba/F3 cells at lower erlotinib concentrations observed in Fig. 5a, b. The key difference appears to be a residual population of EGFR L858R + Y891D that retains signaling capacity even at higher erlotinib concentrations.

One possible explanation for the resistance to early generation EGFR inhibitors seen for EGFR L858R + Y891D in Ba/F3 cell viability assays is that signaling by a minor residual misfolded subset of the receptor population is not efficiently inhibited by erlotinib or afatinib, and that this small uninhibited population is sufficient to promote cell proliferation and viability. Indeed, activation of only a small fraction of EGFR in a cell is required to maintain ERK signaling^38^ and to drive tumorigenesis^31,32^. Accordingly, the majority (at least 80–90%) of the target must be inhibited in order to reduce cell viability (as exemplified in studies of BRAF inhibitors)^39^. We suggest that the misfolded population may have reduced affinity for erlotinib and afatinib (which both require an intact ATP-binding site in the kinase for high-affinity binding) such that it is not well-inhibited at intracellular inhibitor concentrations. The small amount of autophosphorylation afforded by binding to ATP (present at mM concentrations) then drives signaling. This is a similar argument to that made for the ability of EGFR variant III (vIII) to promote transformation despite being reported to have only a low level of constitutive activity^40,41^. In this scenario, one might predict that altered sensitivity to osimertinib is more modest than that seen for erlotinib or afatinib. As a non-covalent inhibitor, erlotinib relies on the proper conformation of the EGFR TKD for its binding to the receptor. Inhibition by the second generation inhibitor afatinib requires non-covalent docking of the inhibitor into an intact binding site prior to reaction with C797. Inhibition by osimertinib (which reacts covalently with C797) may be less susceptible to the destabilized EGFR TKD, and (unlike afatinib) its reactivity with EGFR is not affected by the T790M mutation^42,43^. Osimertinib may retain some ability to react with C797 in a partially misfolded EGFR, as suggested by its known reactivity with a range of cysteines in proteins that have no structural resemblance to EGFR^33^. This is consistent with our observation of persistent EGFR activation for L858R + Y891D at higher concentrations of erlotinib but not of osimertinib (Fig. 5).

Protein misfolding leading to kinase retention and aberrant signaling in the endoplasmic reticulum has been suggested as a mechanistic basis of the activating internal tandem duplication in the receptor tyrosine kinase FLT3 (FLT3-ITD), a recurrent alteration in acute myeloid leukemia^44–46^. In addition, compounds that restore stability and function of the tumor suppressor p53 with a Y220C mutation (which destabilizes its DNA-binding domain) have recently been described, suggesting some alterations that promote misfolding may be actionable^47^. To our knowledge, however, protein misfolding has not previously been suggested as a potential modifier of sensitivity to a targeted therapy. A limitation of our study is the absence of an endogenous model of EGFR L858R + Y891D in which to further evaluate a possible role for protein misfolding in altering TKI sensitivity. We cannot rule out the possibility of alternative mechanistic explanations for the decreased sensitivity of EGFR L858R + Y891D to EGFR TKIs that may be independent of the destabilizing effect of Y891D. Despite this limitation, our data demonstrate that co-occurring or acquired EGFR mutations can modulate sensitivity to targeted therapies through mechanisms other than direct steric hindrance and altered ATP binding affinity and highlight the need to explore the impact of molecular alterations that disrupt folding and stability of oncogenic proteins.

We note that all 4 cases of EGFR Y891D identified to date occur in the context of an activating L858R mutation. To our knowledge, Y891D has not been observed in combination with other activating EGFR alterations such as an exon 19 deletion. Our data suggest that mutating Y891 to aspartate in combination with L858R de-stabilizes EGFR, and it is possible that Y891D in the context of an exon 19 deletion may be even further compromised. Consistent with this, we were unable to express the classic EGFR ∆E746-A750 exon 19 deletion with a Y891D secondary mutation in Ba/F3 cells. Prior studies have demonstrated that some secondary EGFR resistance mutations (such as L718X) may arise preferentially in the context of L858R but not ∆E746-A750^48–50^. These findings suggest that the identity of the activating EGFR mutation may delineate the spectrum of potential secondary mutations that can co-occur or emerge.

In sum, our findings provide biochemical and structural insights about an uncommon co-occurring or acquired EGFR mutation that promotes resistance to first generation EGFR inhibitors through a mechanism distinct from those of other EGFR alterations. Co-occurrence of Y891D with L858R appears to destabilize EGFR without completely disrupting signaling. Our biochemical data argue against altered ATP affinity or steric hindrance to drug binding as mechanisms of EGFR TKI resistance mediated by Y891D and raise the possibility of protein misfolding with preserved signaling as a previously unrecognized etiology of EGFR TKI resistance.

## Methods

### Informed consent and tumor molecular analysis

The study participant provided informed written consent under a research protocol approved by an Institutional Review Board (Yale HIC #1603017333). The study was conducted in accordance with the Declaration of Helsinki. Tumor molecular analysis was performed by the Tumor Profiling Laboratory at Yale-New Haven Hospital using the Oncomine Comprehensive Assay v3 next generation sequencing platform to assay 161 cancer-related genes.

### Cell lines and reagents

Ba/F3 cells were maintained in RPMI-1640 (Gibco) supplemented with 10% heat-inactivated fetal bovine serum (FBS; Gibco), penicillin/streptomycin (Gibco), and 1 ng/ml interleukin-3 (IL-3; Gibco). Mycoplasma testing of Ba/F3 cells was negative. Sf9 cells were maintained and cultured as previously described^16^. Erlotinib, afatinib and osimertinib were obtained from Selleck Chemicals.

### Cloning and site-directed mutagenesis

Total RNA from HEK 293T cells was used as a template for cDNA preparation with oligo-dT primers using the SuperScript III First-Strand Synthesis System (Invitrogen). Full-length wild-type EGFR was PCR-amplified using the Q5 High-Fidelity DNA Polymerase (New England BioLabs) using primers with incorporation of *attB* recombination sites for Gateway cloning (Invitrogen). The forward primer GGGGACAACTTTGTACAAAAAAGTTGGCACC**ATG**CGACCCTCCGGGACGGCC and reverse primer GGGGACAACTTTGTACAAGAAAGTTGGCAA**TCA**TGCTCCAATAAATTCACT were used to amplify EGFR with *attB* recombination sites. PCR products were gel-purified and cloned into pDONR223 (Invitrogen) using BP Clonase (Invitrogen). The L858R mutation was introduced using the Q5 Site-Directed Mutagenesis Kit (New England BioLabs) with the forward primer GATTTTGGGC**G**GGCCAAACTG and reverse primer TGTGATCTTGACATGCTGC. Y891D was then introduced into the EGFR L858R construct with site-directed mutagenesis using the forward primer ACACAGAATC**G**ATACCCACCAG and reverse primer AAAATTGATTCCAATGCCATC. EGFR constructs in pDONR223 were sub-cloned into the lentiviral pLX307 vector using LR Clonase (Invitrogen). All constructs were confirmed by Sanger sequencing. Lentivirus was produced as previously described^51^.

### Ba/F3 transformation and cell viability assays

cDNAs encoding EGFR constructs in pLX307 were introduced into Ba/F3 cells via lentiviral transduction with 8 μg/ml polybrene and centrifugation at 2250 rpm at 37 °C for 30 min. After 24 h, transduced cells were selected with 1 μg/ml puromycin for at least 5 days. Interleukin-3 (IL-3) independent growth was achieved for Ba/F3 cells expressing EGFR L858R and L858R + Y891D by withdrawal of IL-3 from culture media.

For cell viability assays, Ba/F3 cells were seeded in white clear-bottom 96-well microtiter plates. Drug or drug vehicle was added on the same day. Following 3 days of drug exposure, cell viability was determined using the Cell Titer-Glo luminescent assay (Promega) according to the manufacturer’s instructions. Luminescence was measured with a Spectramax M3 plate reader (Molecular Devices). Data analysis was performed using GraphPad Prism software.

### Antibodies and immunoblotting

Antibodies against EGFR (#2239), phospho-EGFR (Tyr 1068; #3777), AKT (#2920), phospho-AKT (Ser 473; #4060), ERK 1/2 (#4695), phospho-ERK 1/2 (Thr 202 / Tyr 204; #9101), and actin (#3700) were obtained from Cell Signaling and used at 1:1000 dilution. HRP-conjugated anti-mouse (#NA931) and anti-rabbit (#NA934) secondary antibodies were obtained from Cytiva and used at 1:10,000 dilution. All samples shown on Western blots were derived from the same experiment and processed in parallel. Uncropped blots corresponding to data presented in Figs. 2e and 5a, c are provided in Supplementary Data (Supplementary Figs. 3–5).

For experiments with EGFR TKI treatment, Ba/F3 cells expressing EGFR L858R or EGFR L858R + Y891D were plated at a density of 1 ×10^6^ cells/ml in 4 ml of media followed by a 2 h incubation. DMSO or drug was then added, and cells were incubated for 4 h followed by collection of cell lysates. For experiments with EGF treatment, Ba/F3 cells expressing EGFR L858R or EGFR L858R + Y891D were serum-starved overnight at a density of 1 × 10^6^ cells/ml in 4 ml of serum-free media. Cells were incubated in serum-free media with or without 100 ng/ml EGF (Gibco; #10450-013) on ice for 10 min. After treatment, cells were washed three times with cold PBS followed by collection of cell lysates.

Cells were lysed using RIPA buffer (Abcam #ab156034) with protease inhibitors (Roche #11836153001) and phosphatase inhibitors (Sigma #P5726, #P0044). Lysates were fractionated by SDS-polyacrylamide gel electrophoresis and transferred to nitrocellulose using the Trans-Blot Turbo transfer system (Bio-Rad). Nitrocellulose membranes were blocked either in Western Blocker Solution (Sigma; #W0138) or EveryBlot Blocking Buffer (Bio-Rad; #12010020). All primary antibody incubations were performed overnight at 4 °C, and secondary antibody incubations were performed for 1 h at room temperature. Chemiluminescence was detected using SuperSignal West Pico PLUS or Femto Chemiluminescent substrate (Thermo Scientific; #34580 and #34096). Images were captured with a ChemiDoc MP Imaging System (Bio-Rad).

### Protein expression and purification

Sf9 cells at 1.6 to 1.8 ×10^6^ cells/ml were infected with recombinant baculovirus and cultured for 60–65 h at 27 °C. Cells were harvested by centrifugation at 2,200 × *g*, and cell pellets resuspended in cold (4 °C) lysis buffer (20 mM Tris/HCl, pH 8.0, containing 500 mM NaCl, 5 mM 2- mercaptoethanol, and 10% w/v glycerol – supplemented with Roche protease inhibitor cocktail). Cells were then lysed using a microfluidizer (Microfluidics M-110P). Cell lysate was centrifuged at 75,000 × *g* for 1 h to remove cell debris and insoluble aggregates, and the resulting supernatant was filtered through a 0.45 μm filter before loading onto a 1.5 ml Ni-NTA affinity column (Qiagen) equilibrated in lysis buffer. The column was washed twice with 20 volumes of ‘wash-1 buffer’ [lysis buffer + 200 mM NaCl] and then ‘wash-2’ [lysis buffer + 15 mM imidazole]. Protein was then eluted with 5 ml of lysis buffer containing 250 mM imidazole. The eluate was filtered (0.22 μm filter) and protein further purified at 4 °C by gel filtration using a HiLoad 16/60 Superdex 200 column (Cytiva) equilibrated in buffer C (20 mM Tris/HCl, pH 7.8, containing 250 mM NaCl, and 250 mM KCl).

### Steady-state kinase assays

Steady-state parameters for kinase activity and inhibition were determined using a quantitative fluorometric peptide assay employing AssayQuant PhosphoSens® peptide substrate #0001 (AQpeptide), containing a sulfonamido-oxine (Sox) fluorophore that shows chelation-enhanced fluorescence upon peptide phosphorylation^26^. The assay reaction (20 μl) contained 50 mM HEPES (pH 7.5), 0.01% Brij-35, 10 mM MgCl_2_, 0.5% w/v glycerol, 0.1 mg/ml BSA, 1 mM DTT, and varying concentrations of AQpeptide, plus the noted concentration of purified recombinant EGFR TKD. Reactions were carried out in a 384-well assay plate format at 30 °C and initiated with the addition of AQpeptide after 5 min incubation of EGFR with ATP. Progress curves of phosphorylated peptide accumulation were monitored using a BioTek Synergy microplate reader with a fluorescence intensity module excitation and emission wavelength at 360 nm and 480 nm, respectively. To convert relative fluorescence intensity readings to molar concentrations of phosphorylated AQpeptide, we fully phosphorylated AQpeptide at a range of different concentrations up to 200 μM and plotted the fluorescence intensity counts of fully phosphorylated AQpeptide against known peptide concentration.

Michaelis constants for ATP (*K*_M, ATP_) were determined for each EGFR variant using 20 μM AQpeptide and a range of different ATP concentrations (0.98 μM–2 mM), with purified EGFR TKDs at the following concentrations: 50 nM (L858R + Y891D), 1 μM (Y891D). Data for the wild-type and L858R-mutated TKD were previously reported^16^.

### Determination of inhibitor IC_50_ values

The half maximal inhibitory concentrations (IC_50_) of erlotinib, afatinib, and osimertinib were determined for each EGFR TKD variant by monitoring reaction progress curves at different concentrations of inhibitor in the presence of 1 mM ATP. Purified L858R + Y891D-mutated TKD at 50 nM was mixed with 0 to 10 μM inhibitor (erlotinib, afatinib, or osimertinib) at 1 mM ATP (and 10 mM MgCl_2_) prior to initiating phosphorylation reactions by adding 10 μM AQPeptide. Fluorescence intensity counts corresponding to phosphorylated AQpeptide were then monitored using the BioTek Synergy 2 microplate reader. Initial velocities were determined as described above, and IC_50_ values were determined by fitting measured velocities (normalized to 100% without inhibitor) using GraphPad Prism to the simple equation: Rate = 100/(1 + [TKI]/IC_50_).

### Statistical analysis

Data were plotted as mean ± SEM from at least 3 independent experiments as indicated in the figure legends. Statistical analysis was performed with unpaired two-tailed Student’s *t* test using GraphPad Prism (version 9). *P* < 0.05 was considered significant.

### Reporting summary

Further information on research design is available in the Nature Research Reporting Summary linked to this article.

### Supplementary information

Supplementary Materials

Reporting Summary

## Data Availability

All data generated or analyzed during this study are included in this published article and its supplementary information files. Requests for materials generated from this study should be directed to the corresponding author.
