# Supplementary material for: A destabilizing Y891D mutation in activated EGFR impairs sensitivity to kinase inhibition
Source: NPJ Precis Oncol. 2024 Jan 5;8:3. doi: 10.1038/s41698-023-00490-w (PMC10770066; doi:10.1038/s41698-023-00490-w)
Supplement: Supplementary file 1 — Supplementary Materials [file 41698_2023_490_MOESM1_ESM.pdf]

# SUPPLEMENTARY DATA

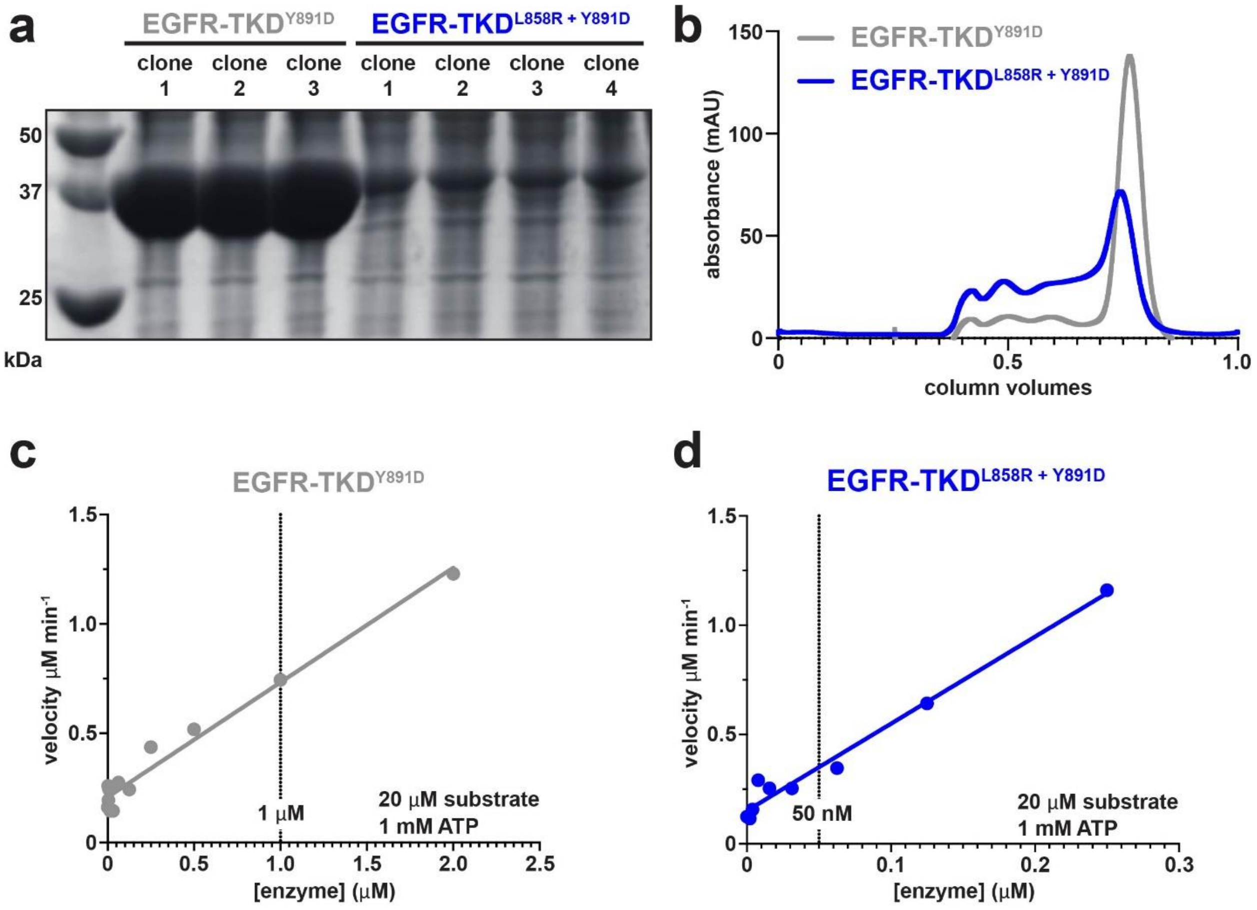


**Supplementary Figure 1.** Expression and activity analysis for EGFR-TKD$^{L858R + Y891D}$
**a** Expression levels of Y891D (EGFR-TKD$^{Y891D}$) and L858R + Y891D (EGFR-TKD$^{L858R + Y891D}$) TKD proteins in test Sf9 infections with P2 virus, with each clone generated from an independent preparation of bacmid DNA. The expected molecular weight for EGFR-TKD is 38k Da. **b** Gel filtration chromatographs of EGFR-TKD$^{L858R + Y891D}$ (blue) and EGFR-TKD$^{Y891D}$ (grey) variants prepared from 4 l of infected Sf9 as described in Methods. EGFR-TKD$^{L858R + Y891D}$ shows a substantially lower level of expression than EGFR-TKD$^{Y891D}$. **c**, **d** Kinase assays showing that the reaction velocity is linear with increasing enzyme concentrations in the ranges used for further kinetic measurements. Since EGFR-TKD$^{L858R + Y891D}$ is activated, whereas EGFR-TKD$^{Y891D}$ shows only wild-type levels of activity, further analysis of EGFR-TKD$^{Y891D}$ was performed at 1 μM enzyme, whereas analysis of (more active) EGFR-TKD$^{L858R + Y891D}$ was performed using 50 nM enzyme.

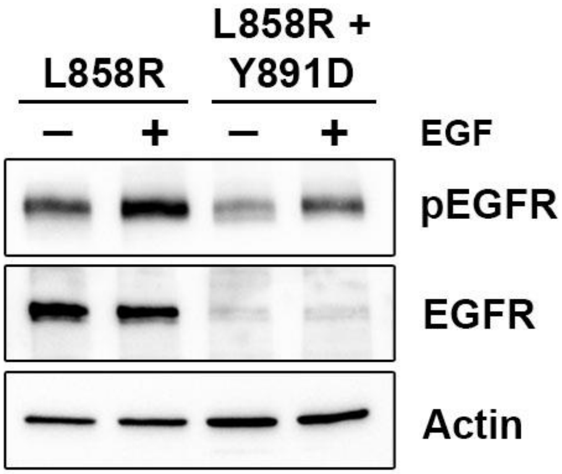


**Supplementary Figure 2.** Cell surface activation of EGFR L858R + Y891D in response to EGF ligand. Ba/F3 cells driven by EGFR L858R or EGFR L858R + Y891D were serum-starved overnight and treated with 100 ng/ml EGF on ice for 10 min. Cell lysates were harvested, and Western immunoblotting was performed with the indicated antibodies.

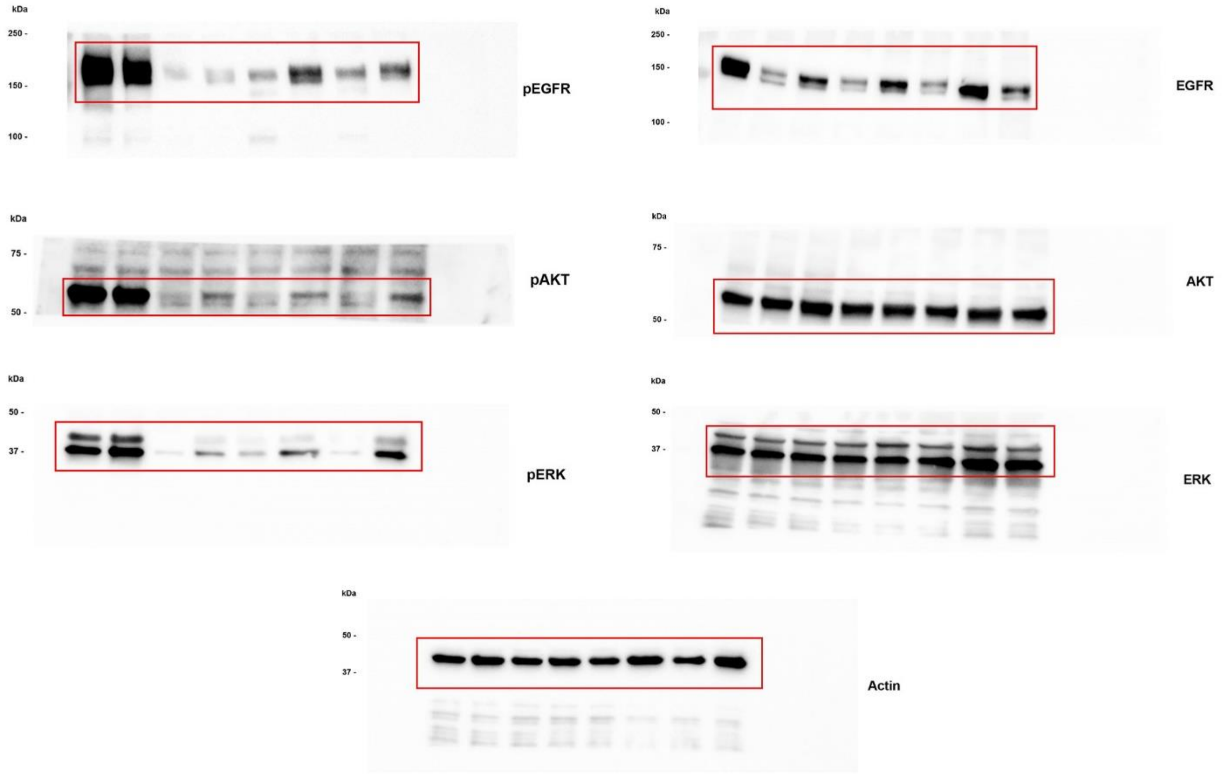


**Supplementary Figure 3.** Uncropped images corresponding to Western blot shown in Figure 2e.

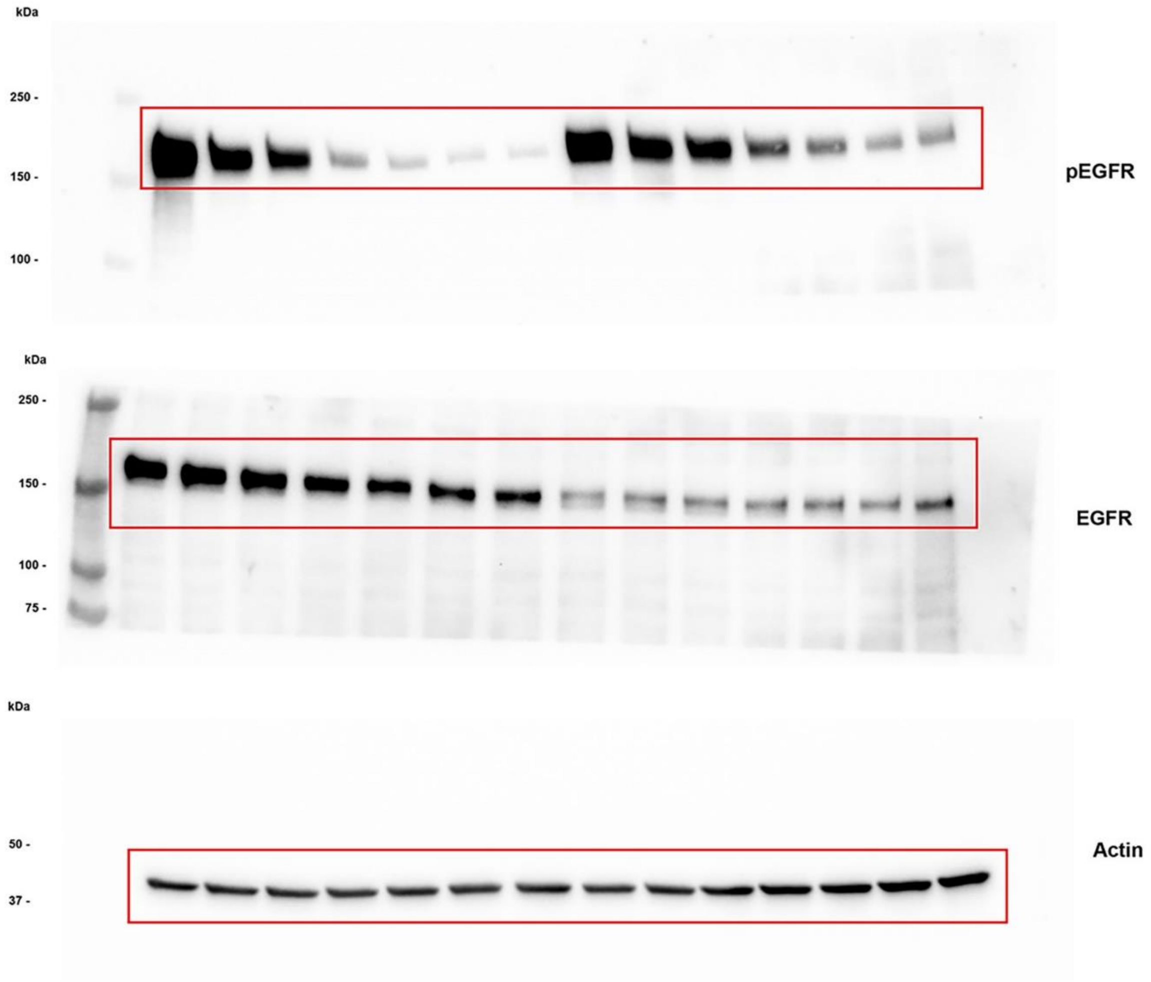


**Supplementary Figure 4.** Uncropped images corresponding to Western blot shown in Figure 5a.

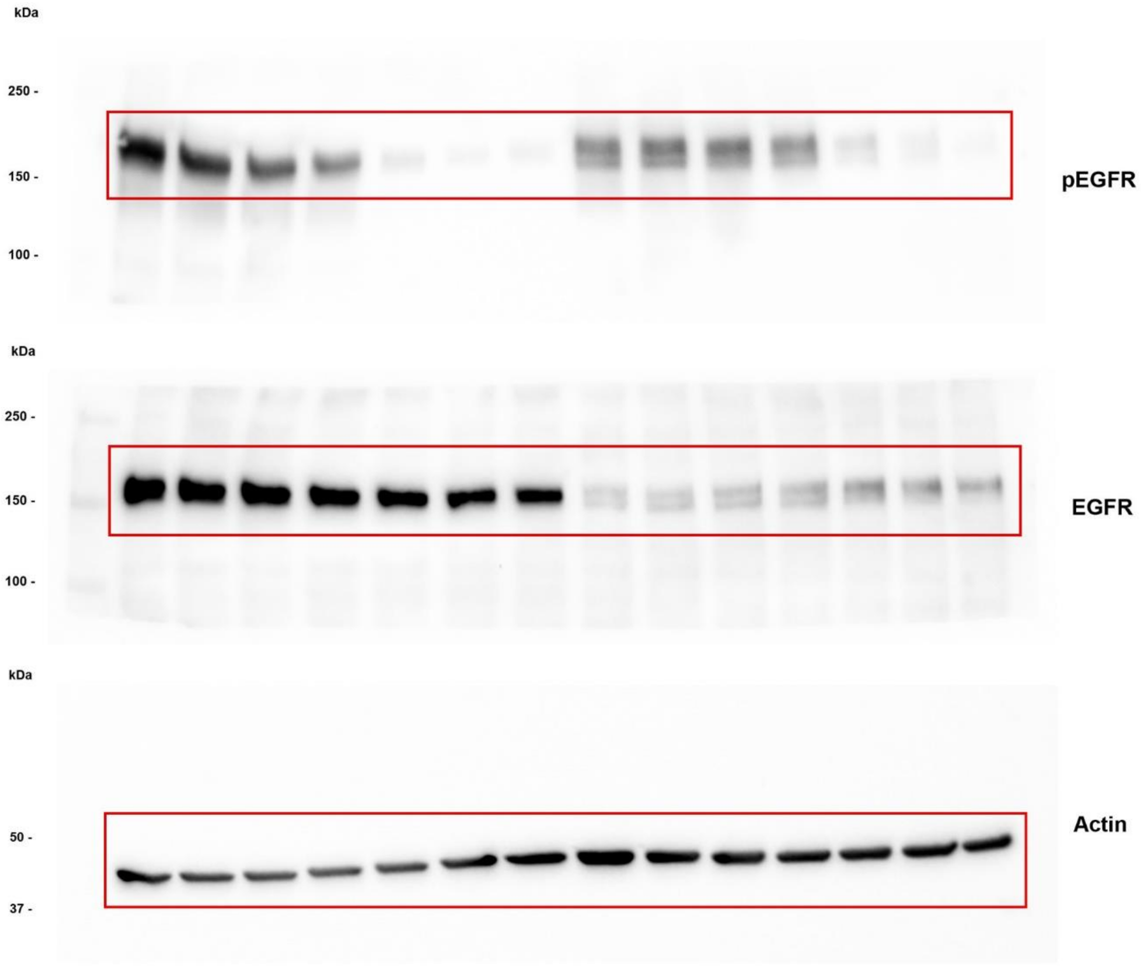


**Supplementary Figure 5.** Uncropped images corresponding to Western blot shown in Figure 5c.
