# Supplementary material for: A destabilizing Y891D mutation in activated EGFR impairs sensitivity to kinase inhibition
Source: NPJ Precis Oncol. 2024 Jan 5;8:3. doi: 10.1038/s41698-023-00490-w (PMC10770066; doi:10.1038/s41698-023-00490-w)
Supplement: Supplementary file 2 — Reporting Summary [file 41698_2023_490_MOESM2_ESM.pdf]

nature portfolio

Corresponding author(s): Wilson

Last updated by author(s): Nov 17, 2023

Reporting Summary

Nature Portfolio wishes to improve the reproducibility of the work that we publish. This form provides structure for consistency and transparency in reporting. For further information on Nature Portfolio policies, see our Editorial Policies and the Editorial Policy Checklist.

Statistics

For all statistical analyses, confirm that the following items are present in the figure legend, table legend, main text, or Methods section.

n/a	Confirmed	
☐	☒	The exact sample size (*n*) for each experimental group/condition, given as a discrete number and unit of measurement
☐	☒	A statement on whether measurements were taken from distinct samples or whether the same sample was measured repeatedly
☐	☒	The statistical test(s) used AND whether they are one- or two-sided<br>*Only common tests should be described solely by name; describe more complex techniques in the Methods section.*
☒	☐	A description of all covariates tested
☒	☐	A description of any assumptions or corrections, such as tests of normality and adjustment for multiple comparisons
☒	☐	A full description of the statistical parameters including central tendency (e.g. means) or other basic estimates (e.g. regression coefficient) AND variation (e.g. standard deviation) or associated estimates of uncertainty (e.g. confidence intervals)
☒	☐	For null hypothesis testing, the test statistic (e.g. *F*, *t*, *r*) with confidence intervals, effect sizes, degrees of freedom and *P* value noted<br>*Give P values as exact values whenever suitable.*
☒	☐	For Bayesian analysis, information on the choice of priors and Markov chain Monte Carlo settings
☒	☐	For hierarchical and complex designs, identification of the appropriate level for tests and full reporting of outcomes
☒	☐	Estimates of effect sizes (e.g. Cohen's *d*, Pearson's *r*), indicating how they were calculated

Our web collection on statistics for biologists contains articles on many of the points above.

Software and code

Policy information about availability of computer code

Data collection	No software was used
Data analysis	No software was used

For manuscripts utilizing custom algorithms or software that are central to the research but not yet described in published literature, software must be made available to editors and reviewers. We strongly encourage code deposition in a community repository (e.g. GitHub). See the Nature Portfolio guidelines for submitting code & software for further information.

Data

Policy information about availability of data

All manuscripts must include a data availability statement. This statement should provide the following information, where applicable:

- Accession codes, unique identifiers, or web links for publicly available datasets
- A description of any restrictions on data availability
- For clinical datasets or third party data, please ensure that the statement adheres to our policy

All data generated or analyzed during this study are included in this published article and its supplementary information files.

Research involving human participants, their data, or biological material

Policy information about studies with human participants or human data. See also policy information about sex, gender (identity/presentation), and sexual orientation and race, ethnicity and racism.

Reporting on sex and gender	This study involved a single male participant.
Reporting on race, ethnicity, or other socially relevant groupings	The study participant was Caucasian.
Population characteristics	The case history includes additional relevant participant characteristics.
Recruitment	The participant was recruited as part of a project focusing on human subjects with advanced non-small cell lung carcinoma.
Ethics oversight	Yale University Institutional Review Board

Note that full information on the approval of the study protocol must also be provided in the manuscript.

Field-specific reporting

Please select the one below that is the best fit for your research. If you are not sure, read the appropriate sections before making your selection.

☒ Life sciences ☐ Behavioural & social sciences ☐ Ecological, evolutionary & environmental sciences

For a reference copy of the document with all sections, see nature.com/documents/nr-reporting-summary-flat.pdf

Life sciences study design

All studies must disclose on these points even when the disclosure is negative.

Sample size	Cell-based assays were performed using at least 3 biological replicates and were independently performed at least 3 times.
Data exclusions	No data were excluded from the analyses.
Replication	All attempts at replication were successful.
Randomization	Samples were randomly allocated to experimental treatment groups.
Blinding	Due to experimental approaches required to conduct the assays, investigators were not blinded to study groups.

Reporting for specific materials, systems and methods

We require information from authors about some types of materials, experimental systems and methods used in many studies. Here, indicate whether each material, system or method listed is relevant to your study. If you are not sure if a list item applies to your research, read the appropriate section before selecting a response.

Materials & experimental systems

n/a	Involved in the study	
☐	☒	Antibodies
☐	☒	Eukaryotic cell lines
☒	☐	Palaeontology and archaeology
☒	☐	Animals and other organisms
☒	☐	Clinical data
☒	☐	Dual use research of concern
☒	☐	Plants

Methods

n/a	Involved in the study	
☒	☐	ChIP-seq
☒	☐	Flow cytometry
☒	☐	MRI-based neuroimaging

Antibodies

Antibodies used	Antibodies against EGFR (#2239), phospho-EGFR (Tyr 1068; #3777), AKT (#2920), phospho-AKT (Ser 473; #4060), ERK 1/2 (#4695), phospho-ERK 1/2 (Thr 202 / Tyr 204; #9101), and actin (#3700) were obtained from Cell Signaling and used at 1:1000 dilution. HRP-conjugated anti-mouse (#NA931) and anti-rabbit (#NA934) secondary antibodies were obtained from Cytiva and used at 1:10,000 dilution.

Validation	Western immunoblotting was performed to confirm identification of proteins of the expected size.

Eukaryotic cell lines

Policy information about cell lines and Sex and Gender in Research

Cell line source(s)	Ba/F3 cells (RRID: CVCL_0161) were provided by the laboratory of Dr. Matthew Meyerson (Dana-Farber Cancer Institute, Boston, MA)
Authentication	Ba/F3 cells were confirmed to require supplemental IL3 for proliferation.
Mycoplasma contamination	Cells tested negative for mycoplasma contamination
Commonly misidentified lines (See ICLAC register)	No commonly misidentified lines were utilized.
